# Antifungal effects of volatile organic compounds produced by *Trichoderma koningiopsis* T2 against *Verticillium dahliae*

**DOI:** 10.3389/fmicb.2022.1013468

**Published:** 2022-09-21

**Authors:** Wei-Liang Kong, Hang Ni, Wei-Yu Wang, Xiao-Qin Wu

**Affiliations:** ^1^Co-innovation Center for Sustainable Forestry in Southern China, College of Forestry, Nanjing Forestry University, Nanjing, China; ^2^Jiangsu Key Laboratory for Prevention and Management of Invasive Species, Nanjing Forestry University, Nanjing, China

**Keywords:** *Trichoderma koningiopsis*, volatile organic compounds, melanin, microsclerotia, *Verticillium dahliae*

## Abstract

Volatile organic compounds (VOCs) produced by microorganisms are considered promising environmental-safety fumigants for controlling soil-borne diseases. *Verticillium dahliae*, a notorious fungal pathogen, causes economically important wilt diseases in agriculture and forestry industries. Here, we determined the antifungal activity of VOCs produced by *Trichoderma koningiopsis* T2. The VOCs from *T. koningiopsis* T2 were trapped by solid-phase microextraction (SPME) and tentatively identified through gas chromatography–mass spectrometry (GC/MS). The microsclerotia formation, cell wall-degrading enzymes and melanin synthesis of *V. dahliae* exposed to the VOC mixtures and selected single standards were examined. The results showed that the VOCs produced by strain T2 significantly inhibited the growth of *V. dahliae* mycelium and reduced the severity of *Verticillium* wilt in tobacco and cotton. Six individual compounds were identified in the volatilome of *T. koningiopsis* T2, and the dominant compounds were 3-octanone, 3-methyl-1-butanol, butanoic acid ethyl ester and 2-hexyl-furan. The VOCs of strain T2 exert a significant inhibitory effect on microsclerotia formation and decreased the activities of pectin lyase and endo-β-1,4-glucanase in *V. dahliae*. VOCs also downregulated the *VdT3HR*, *VdT4HR*, and *VdSCD* genes related to melanin synthesis by 29. 41-, 10. 49-, and 3.11-fold, respectively. Therefore, *T. koningiopsis* T2 has potential as a promising biofumigant for the biocontrol of *Verticillium* wilt disease.

## Introduction

*Verticillium dahliae* is a soil-borne plant pathogen that exists worldwide and causes vascular wilts in more than 38 families of plants including 660 species, ranging from herbaceous annuals to woody perennials, such as *Gossypium* spp., *Lycopersicon esculentum*, *Solanum tuberosum*, *Fragaria* × *ananassa*, *Canarium album*, *Chaenomeles japonica*, and *Ulmus pumila* (Giovanni et al., 2019; Hanson and Radionenko, 2021; Van Eerd et al., 2021; Zhang G. L. et al., 2021; Godena et al., 2022). Once infected with the pathogen, plants exhibit wilting, yellowing, necrosis, vascular discoloration, parietal leaf curling, dwarfing, wilting and premature senescence, resulting in great ecological damage and economic losses to agriculture and forestry (Kowalska, 2021; Liu L. S. et al., 2021; Lu et al., 2021).

Due to the stable dormant structure microsclerotia, long-term variability, and coevolution with host plants (Ju et al., 2020; Höfer et al., 2021; Starke et al., 2021), controlling the spread of *Verticillium* wilt in plants remains challenging using currently available methods, such as chemical control, genetic breeding, and optimization of the cropping pattern (Ostos et al., 2020; Ramirez-Gil and Morales-Osorio, 2021; Zhang Y. L. et al., 2021). Disease resistance breeding is limited by a long breeding cycle and a lack of natural resistance resources, and chemical control has no beneficial effect on the control of soil-borne diseases (Diaz-Rueda et al., 2021; Hanika et al., 2021; Sunico et al., 2022). With the social pressure to make agriculture more sustainable while maintaining a healthy environment, the identification of new strategies that can reduce the use of chemicals is necessary.

*Trichoderma* is well known as a biological control agent (BCA) that is used to manage diseases in a wide variety of plants, and several *Trichoderma* species have been commercially developed as biofungicides and biofertilizers (Abo-Elyousr and Almasaudi, 2022; El-Komy et al., 2022; Olowe et al., 2022). Different isolates of *Trichoderma* spp. are being successfully used and commercialized to combat a wide range of soil phytopathogenic fungi, such as *Fusarium oxysporum*, *Rhizoctonia solani*, *Sclerotium rolfsii*, *S. cepivorum*, and *Alternaria alternate* (Rabinal and Bhat, 2020; Godara and Singh, 2021; Khalil et al., 2021; Álvarez-García et al., 2022). The mechanisms of *Trichoderma*-based plant disease biocontrol include their capacity for nutrient and space competition, parasitism, secretion of antimicrobial metabolites (Cubilla-Ríos et al., 2019; da Silveira et al., 2021; Kamaruzzaman et al., 2021), activation of defense responses, and promotion of plant growth (Zehra et al., 2017; Chen et al., 2021). Among these approaches, the application of VOCs is considered a promising biocontrol strategy for the management of plant diseases, particularly soil-borne diseases.

Fungal VOCs are a large group of carbon-based chemicals with low molecular weights, low polarities, low boiling points, and high vapor pressure (Jiang et al., 2021). These characteristics provide VOCs with advantages over other secondary metabolites of a microbial nature, and these advantages include the abilities to travel long distances, to mediate interactions between organisms that are not in direct contact and to be perceived at low concentrations (Yusnawan et al., 2021). Some biocontrol strains emit VOCs with antibacterial and antifungal activities and thus have the potential to affect biocontrol activities at a distance (Alvarez-Garcia et al., 2020; Ruangwong et al., 2021b). For instance, VOCs produced by the biocontrol strain *Bacillus amyloliquefaciens* SQR-9 influence the growth and virulence traits of the tomato wilt pathogen *Ralstonia solanacearum* (Raza et al., 2016). *Pseudomonas chlororaphis* subsp. *aureofaciens* SPS-41 can emit VOCs to control *Ceratocystis fimbriata* in postharvest sweet potato. Recent studies showed that allyl isothiocyanate in the volatiles of *Brassica juncea* inhibits the growth of root rot pathogens in *Panax notoginseng* by inducing the accumulation of reactive oxygen species (ROS) (Zhang et al., 2019; Liu H. J. et al., 2021). Thus, the exploitation of the biofumigation properties of microbial VOCs promises to be a novel and ecologically friendly approach for the control of soil-borne diseases.

In a previous study, we found that the VOCs produced by *Trichoderma koningiopsis* T2 exert a strong antagonistic effect on *Colletotrichum gloeosporioides* and *Alternaria alternata* (Kong et al., 2022). However, whether the VOCs produced by this fungus can inhibit other pathogens and the types of VOCs released remain unclear. The aims of this study were to clarify the antagonistic mode of VOCs produced by strain T2 against *V. dahliae*, to identify individual VOCs associated with inhibiting the growth of *V. dahliae* and to provide reference data and technical support for the management of plant *Verticillium* wilt.

## Materials and methods

### Fungal strains and growth media

*Trichoderma koningiopsis* T2, which was previously isolated from the leaves of *Liriodendron chinense* × *tulipifera*, was used in this study (Kong et al., 2022). The target pathogen *V. dahliae* At13 was isolated from *Acer truncatum* Bunge in Shandong Province, China (Li et al., 2018). Both strains were routinely cultured on potato dextrose agar medium (PDA; Difco-Becton, Dickinson, Sparks, MD, USA).

### Effect of *Trichoderma* volatile organic compounds on the mycelial growth of *Verticillium dahliae*

The antifungal activity of VOCs produced by *T. koningiopsis* T2 was detected after culturing on two sealed Petri dishes (Kong et al., 2020a). Each Petri dish contained 20 mL of PDA medium, and a 6-mm-diameter plug of strain T2 was placed on the center of one of the PDA plates. *V. dahliae* had been inoculated on the 20 mL PDA 4 days prior to placing them with *Trichoderma*. The bottoms of the two Petri dishes were sealed with parafilm and cultured in a constant-temperature incubator at 25°C for 10 days. When the fungal hyphae in the control group reached the edge of the Petri dish, two perpendicular diameters of each colony were measured with a vernier caliper. The following formula was used: inhibition rate = [(Cd-6)-(Td-6)] × 100%/(Cd-6), where Cd is the colony diameter on the control PDA plate and Td is the colony diameter on the treated PDA plate. The experiment was repeated twice, and each treatment was conducted in triplicate.

### Experiment on interaction between *Trichoderma* volatiles and plants

Undamaged cotton and tobacco seeds that were uniform in size were surface-sterilized (soaked in 70% ethanol for 2 min and 3% NaClO for 10 min), washed thoroughly with sterile distilled water and air-dried to remove any surface water. The seeds were then sown in 1/2 MS agar medium and vernalized for 2 days at 4°C in the dark. The seeds were then placed in a growth chamber under a long photoperiod (16-h light/8-h dark) and 70% relative humidity at 22°C. The germinated seedlings were arranged in tissue culture bottle that contained sterilized mixed matrix (peat soil: vermiculite: perlite = 5:1:1, V/V), and simultaneously, the cover of a centrifuge tube cultured with *Trichoderma* was placed near the base of the stem of the seedlings to avoid direct physical contact between *Trichoderma* and plants. Then, 5 mL of *V. dahliae* spore suspension (1 × 10^7^ cfu/ml) was inoculated into the roots of each plant. Bottles not treated with *Trichoderma* VOCs were used as control. After 20 days, the occurrence of diseases was observed. The experiment was replicated twice with 10 seedlings in each replicate.

### Detection of the colonization of *Verticillium dahliae* in plant roots

The biomass of *V. dahliae* in cotton and *N. benthamiana* treated or not treated with *T. koningiopsis* T2 VOCs under AT13 infection was estimated by RT–qPCR. DNA was extracted from the roots using a *SteadyPure* Plant Genomic DNA Extraction Kit (AG21011, Accurate Biology, Hunan, China) and was quantified by spectrophotometry, and 50 ng of DNA of each sample was used for RT–qPCR. RT–qPCR was performed using genomic DNA as the template and the Hieff RT–qPCR SYBR Green Master Mix (Yeasen, Shanghai, China) under the following conditions: an initial denaturation step of 95°C for 5 min followed by 40 cycles of 95°C for 10 s and 60°C for 30 s. *V. dahliae* elongation factor 1-α (*VdEF-1*α) was used for the quantification of fungal colonization. The cotton 18S gene and *N. benthamiana EF-1*α were used as the endogenous plant reference gene. The primers are listed in Table 1 (Geng et al., 2022).

**TABLE 1:** Primers for measuring the relative fungal biomass in tobacco and cotton.

Gene name	Primer (5′–3′)
18S-F	CGGCTACCACATCCAAGGAA
18S-R	TGTCACTACCTCCCCGTGTCA
*NbEF-1*α*-F*	AGGATACAACCCTGACAAGA
*NbEF-1*α*-R*	GTGGGACCAAAAGTAACAAC
*VdEF-1*α*-F*	TGAGTTCGAGGCTGGTATCT
*VdEF-1*α*-R*	CACTTGGTGGTGTCCATCTT

### Gas chromatography–mass spectrometry analysis of volatile organic compounds produced by strain T2

Ten 6-mm plugs from strain T2 were placed in a 250-mL Erlenmeyer flask containing 100 mL of liquid PDB medium and cultured in a shaker at 25^°^C and 150 rpm for 7 days. The PDB liquid medium without fungal inoculation was used as a control. To prevent the VOCs from escaping, the conical flask was sealed with tin foil. In this experiment, a 65-μm PDMS/DVB fiber tip was selected to determine the fungal VOCs, and the extraction tip was aged before its first use. The pretreatment temperature of the extraction head selected in this experiment was 250^°^C, and the pretreatment duration was 30 min. The cultured fungal sample was shaken and placed in a water bath at 40^°^C. The needle of the aged SPME was inserted through the tin foil. The sample was adsorbed and extracted for 30 min. After the extraction, the fiber head was rapidly retracted, and the needle was removed and immediately inserted into the gasification chamber in the gas chromatograph (Agilent 7000B, USA). The fiber was pushed out, and the high temperature of the gasification chamber was used to thermally analyze the target object for 3 min.

The GC–MS conditions were as follows: Rtx–5 quartz capillary column; helium was the carrier gas; 230°C was the inlet temperature; the temperature was maintained at the initial temperature of 40°C for 3 min, increased at 10^°^C/min to 95^°^C, increased at 30°C/min to 230°C and maintained at 230^°^C for 5 min; EI source as the ion source; electron energy of 70 eV; and the spectrum search was conducted using Nist 05 and the Nist 05s library (Kong et al., 2020b).

### Effect of commercial volatile compounds on fungal growth

To test the effect of commercial volatile compounds against *V. dahliae*, the sealed plate method was conducted as described above. The commercial volatile compounds were purchased from Rongshide Trading Co., Ltd. (Nanjing, China), and used in the test. The final concentrations varying from 60 to 1300 μL/L of the airspace in a Petri dishes (70 mm-diameter), and *V. dahliae* was separately inoculated as a control. The bioassay plates were incubated at 25°C for 10 day. Three replicates of each treatment were included, and the experiment was repeated twice. The colony diameters of the plant pathogens were measured, and the percentage inhibition was calculated as described above.

### Effect of T2 volatile organic compounds on microsclerotia formation of *Verticillium dahliae*

Ten *V. dahliae* plugs were inoculated in 100 mL of liquid complete medium (CM) (6 g/L yeast extract, 6 g/L acid-hydrolyzed casein and 10 g/L sucrose) and shaken at 25°C for 48 h in a shaker at 150 rpm to produce spores. The mycelium was filtered through sterile gauze, and the fresh spore suspension was diluted with hemocytometer to obtain a concentration of 1 × 10^6^ cfu/mL (Cheng et al., 2017). One hundred microliters of the spore suspension were plated on modified oat medium (20 g/L oatmeal and 20 g/L glucose) to produce microsclerotia (Frederick et al., 2017). The inhibitory effect of the VOC mixture and 10 μL of four types of pure components was detected by the sealed plate method. Fourteen days later, the formation of microsclerotia was observed by a stereomicroscope and record the number in each field of view (SteREO Discovery V.20, Wetzlar, Germany).

### Pectin lyase and endo-β-1,4-glucanase activity assay

After the mycelium of *V. dahliae* was exposed to VOCs for 10 days, 0.05 g of mycelium was weighed and frozen with liquid nitrogen. The mortar and pestle were baked at 180°C for 2 h and then placed into an ultralow-temperature refrigerator (-80°C). The hyphae were ground into powder with a pestle, homogenized with 1 mL of phosphate-buffered saline (PBS) buffer (0.01 M, pH 7.2–7.4) and centrifuged (5,000 rpm, 5 min). The supernatant was then obtained and stored at 4°C. The activities of pectin lyase and endo-β-1,4-glucanase were determined by enzyme-linked immunosorbent assay (ELISA) according to the manufacturer’s instructions (Wuhan Laboratory One Stop Service, China). The experiment was repeated twice, and each treatment was conducted in triplicate.

### RNA extraction and RT–qPCR analysis of pathogenic mycelium

Ten mycelium plugs of *V*. *dahliae* was inoculated in 100 mL of CM medium and shaken at 150 rpm and 25^°^C for 48 h. The suspension was filtered through monolayer gauze and diluted to obtain a spore suspension of 10^6^ cfu/mL. One hundred microliters of the conidia suspension were spread on a PDA plate covered with sterile cellophane to collect mycelium more conveniently. A 6-mm plug of strain T2 was placed on another PDA plate, the mode of co-culture is similar to that described in section “Effect of trichoderma volatile organic compounds on the mycelial growth of *Verticillium dahliae*.” Conidia on PDA treated without T2 VOCs were used as a control. After culture in the dark at 25°C for 48 h, the hyphae of the pathogens in the different treatments were collected, and RNA was extracted using the TRIzol reagent according to the manufacturer’s instructions. After DNase I treatment, 1 μg of ribonucleic acid was added to the 20-μL reaction system, and first-strand cDNA was synthesized using the reverse transcription system according to the manufacturer’s instructions (Kong et al., 2021).

The relative expression levels of the *VdT3HR*, *VdSCD*, and *VdT4HR* genes were determined, and the β-tubulin gene was used as a reference. The expression levels of related genes were calculated using ABI 7500 software (Applied Biosystems, USA) and the 2^–ΔΔCT^ method (Xiong et al., 2014). The primers used to amplify these genes are listed in Table 2.

**TABLE 2:** Primers for detecting melanin gene expression of *Verticillium dahliae.*

Gene name	Primer (5′–3′)	Functions
*VdT3HR*	GAAGGGTGTGACTGTCAATG	Anthocyanin reductase
	TTGATCCACTCGCAGTCTTC	
*VdSCD*	GAGTTCCTCGCCATGATCTC	Cylindrosporone dehydratase
	TACTCGCTCCAACGAATCTC	
*VdT4HR*	AGGTCGTACAAGCCATCAAG	1,3,6,8-THN reductase
	CTCGCGTGTTGATGTTGAAG	
β-tubulin	TTTCCAGATCACCCACTCC	Reference gene
	ACGACCGAGAAGGTAGCC	

### Extraction and quantitative analysis of melanin in *Verticillium dahliae*

Using the method reported by Bashyal et al. (2010), melanin in *V. dahliae* after treatment with and without T2 VOCs was extracted and quantitatively analyzed. The dried hyphae obtained after 10 days of culture were weighed to obtain samples with the same weight. The mycelium was placed in a 50-mL centrifuge tube, 10 mL of distilled water was added, the water bath was boiled for 5 min, and the samples were centrifuged at 11,000 rpm for 5 min, washed and re-centrifuged. Subsequently, 10 mL of NaOH (1 M) was added, and the samples were boiled at 120^°^C in a high-pressure steam sterilizer for 20 min, cooled to adjust the pH to 2.0 to obtain a precipitate, dissolved with NaOH (1 M), centrifuged at 11,000 rpm for 15 min, washed three times with distilled water, and dried in an oven at 85°C. The extracted dried product was dissolved in 5 mL of NaOH (1 M) and centrifuged at 11,000 rpm for 10 min. The supernatant was transferred to a new clean centrifuge tube, and the OD value at 400 nm was measured. The methods described by Ma et al. (2017) were used to calculate the melanin content (g/L) = OD400 × 0.105 × N (N represents the dilution factor). The experiment was repeated twice, and each treatment was conducted in triplicate.

### Statistical analysis

The data were generated by analysis of variance and Duncan’s multiple comparison using SPSS 22.0 software (IBM Inc., Armonk, NY, United States), or Independent Samples *t*-test to determine significant differences (*P* < 0.05). Graphs were drawn using GraphPad Prism 8.0 (GraphPad Software, Inc., United States).

## Results

### Volatile organic compounds produced by *Trichoderma koningiopsis* T2 inhibited the mycelial growth of *Verticillium dahliae* and reduced the severity of *Verticillium* wilt

**FIGURE 1:**
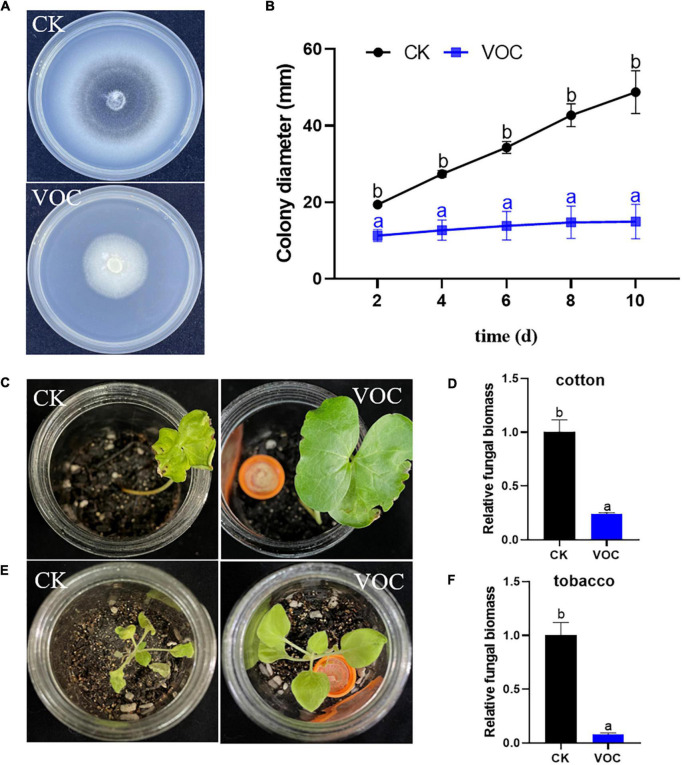
**(A,B)** Effect of *T. koningiopsis* T2 VOCs on the morphology and diameter of *V. dahliae* colonies. **(C,E)** Effect of VOCs on the occurrence of *Verticillium* wilt on tobacco and cotton after 20 days. **(D,F)** Detection of the development of the fungal biomass of *V. dahliae* in plant roots under different treatments. Data are the means ± SE. Different lowercase letters indicate significant differences (*p* < 0.05) among the treatments (*t*-test).

The mycelial growth inhibition assay showed that VOCs produced by *T. koningiopsis* T2 exerted a significant inhibitory effect in the tested conditions. The colony diameter of *V. dahliae* treated with strain T2 VOCs was significantly lower than that of the control, and treatment with the VOCs of strain T2 completely inhibited melanin production in *V. dahliae* (Figure 1A). The quantitative results indicated that the mycelium diameter of *V. dahliae* exposed to the VOCs of strain T2 almost did not increase at the five time points tested; in the absence of VOCs, the mycelium growth of the control increased gradually over time, and the relative rate of inhibition reached 70.59% after 10 days of culture (Figure 1B).

According to the analysis of disease on different hosts, the leaves of cotton and tobacco in the control group showed yellowing, wilting and shrinkage, whereas the plants treated with VOCs exhibited strong growth (Figures 1C,E). The results from the RT–qPCR-based detection of pathogen colonization were consistent with the pathogenic phenotype. The biomass of *V. dahliae* AT13 in tobacco and cotton roots in the untreated control was 4.12- and 12.35-fold higher than that obtained after treatment with *Trichoderma* VOCs, respectively (Figures 1D,F), which indicated that *Trichoderma* VOCs could effectively prevent the colonization of *V. dahliae* on tobacco and cotton and thus reduce the occurrence of *Verticillium* wilt.

### Gas chromatography–mass spectrometry/mass spectrometry analysis of volatile organic compounds produced by *Trichoderma koningiopsis* T2

**FIGURE 2:**
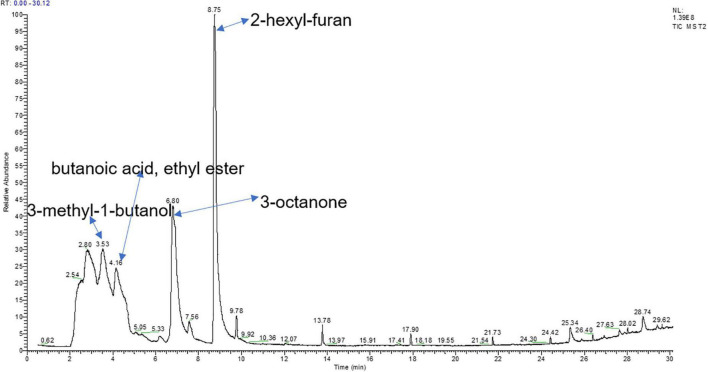
Total ion chromatogram of VOCs from *T. koningiopsis* T2 identified through gas chromatography–mass spectrometry (GC/MS) analysis.

The same volatiles produced by PDA medium were filtered out. A total of 6 compounds with a relative peak area >0.5% were tentatively identified through an NIST library search (Table 3). The most dominant volatile compound detected in this study was 2-hexyl-furan at 8.74 min with a peak area of 39.91%, followed by 3-octanone at 6.79 min with a peak area of 22.73% and ethanolamine at 2.33 min with a peak area of 8.01% (Figure 2). To demonstrate their potential biological activity, we purchased the pure standards presented in Table 2 and tested their antagonistic activity against *V. dahliae*.

**TABLE 3:** GC–MS/MS VOC profile of strain T2.

Retention time (min)	Relative peak area (%)	CAS#	Compound
2.33	8.01	141-43-5	Ethanolamine
3.53	6.41	123-51-3	3-methyl-1-butanol
4.14	3.91	105-54-4	Butanoic acid ethyl ester
6.79	22.73	106-68-3	3-octanone
7.56	2.20	10198-23-9	Cyclohexanol, 1-methyl-4-(1-methylethenyl)-acetate
8.74	39.91	3777-70-6	2-hexyl-furan

### Antifungal activity of commercial volatile compounds against *Verticillium dahliae*

**FIGURE 3:**
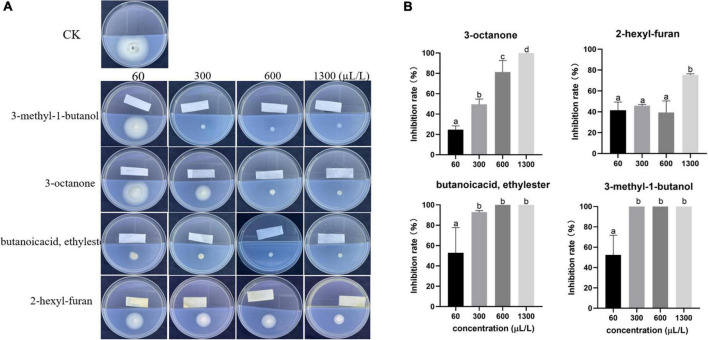
Inhibitory effect of commercial standards at different volumes (60, 300, 600 and 1300 μL/L) on *V. dahliae.* **(A)** Phenotype and **(B)** percentage inhibition. The vertical bars represent the standard deviations of the averages. Different lowercase letters indicate significant differences (*p* < 0.05).

None of the standards with the exception of 3-octanone, 3-methyl-1-butanol, butanoic acid ethyl ester, and 2-hexyl-furan exhibited antifungal activity. These four standards were diluted to different concentrations and cocultured with *V. dahliae* in a divided plate to detect their antagonistic activity. In the concentration range of 60–1,300 μL/L, all four standards easily became volatile and inhibited mycelium growth. Among these standards, 3-octanone (1,300 μL/L), butanoic acid ethyl ester (600 μL/L) and 3-methyl-1-butanol (300 μL/L) completely inhibited the growth of *V. dahliae*, whereas the inhibitory effect of 2-hexyl-furan was weak; even high concentrations (1,300 μL/L) could not completely inhibit the mycelial growth of *V. dahliae* (Figure 3).

### *Trichoderma koningiopsis* T2 volatile organic compounds inhibited the microsclerotia formation of *Verticillium dahliae*

**FIGURE 4:**
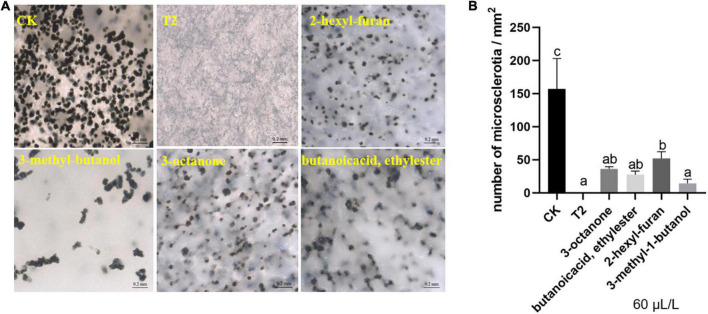
Effect of strain T2 VOCs on microsclerotia formation of *V. dahliae*. **(A)** Phenotypic observation and **(B)** quantitative analysis. The bars in **(A)** represent 200 μm. Different lowercase letters indicate significant differences (*p* < 0.05).

After culture on oat medium for 14 days, the microsclerotia of the control covered the visual field, but the hyphae exposed to the mixture VOCs remained white, and no microsclerotia was detected. At a low concentration (60 μL/L), the four compounds also relatively inhibited the formation of microsclerotia (Figure 4A). Similarly, the statistical results showed that the density of microsclerotia on the control medium was close to 150/mm^2^. In addition, the treatment with commercial standards yielded slightly different results, and among these, 3-methyl-1-butanol exerted the best inhibitory effect, with a density of approximately 15/mm^2^ (Figure 4B).

### *Trichoderma koningiopsis* T2 volatile organic compounds reduced the activity of cell wall-degrading enzymes of *Verticillium dahliae*

**FIGURE 5:**
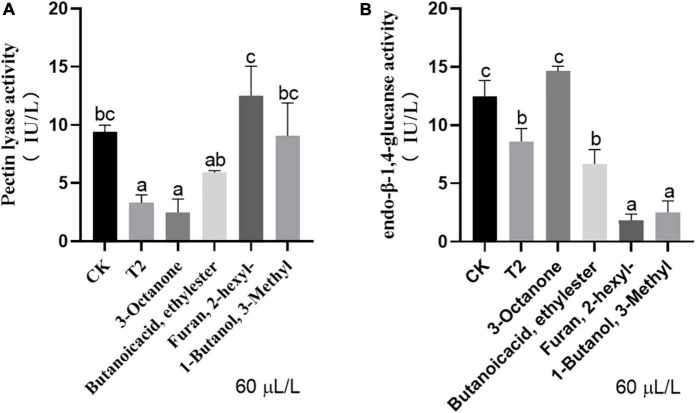
Activities of **(A)** pectin lyase and **(B)** endo-β-1,4-glucanase of *V. dahliae* under different treatments. Vertical bars represent the standard deviation of the average. Different lowercase letters represent significant differences (*p* < 0.05).

The VOC mixture and individual commercial standards exert different effects on the activity of different cell wall-degrading enzymes of *V. dahliae*. For pectinase, 60 μL of 3-octanone exerted the same inhibitory effect as the mixture of VOCs, but no significant difference was found among the other three pure products and the control (Figure 5A). With respect to endo-β-1,4-glucanase, 2-hexyl-furan and 3-methyl-1-butanol exerted the strongest inhibitory effect, and this effect was even stronger than that of the mixture of VOCs. The inhibitory effect of butanoic acid ethyl ester was similar to that of the VOC mixture, whereas 3-octanone had no significant inhibitory effect on endo-β-1,4-glucanase production (Figure 5B).

### *Trichoderma koningiopsis* T2 volatile organic compounds inhibited the formation of melanin in *Verticillium dahliae*

**FIGURE 6:**
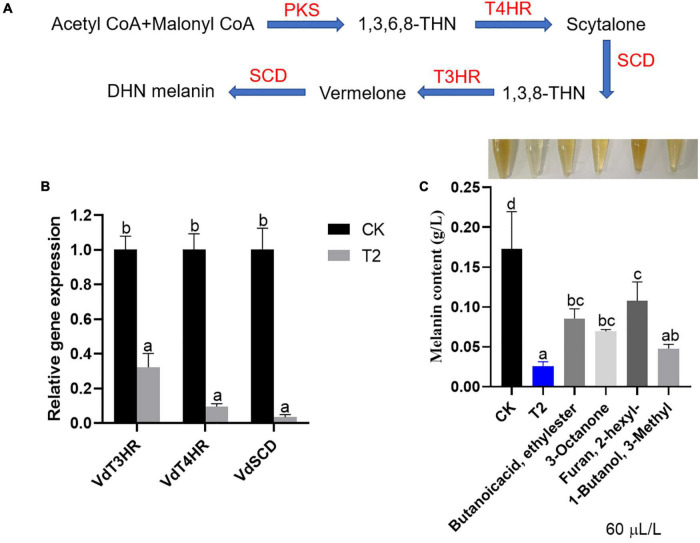
Effect of *T. koningiopsis* T2 VOCs on melanin formation in *V. dahliae*. **(A)** Melanin synthesis pathway, **(B)** relative gene expression levels and **(C)** quantitative analysis of melanin. The vertical bars represent the standard deviations of the averages. Different lowercase letters indicate significant differences (*p* < 0.05).

As shown in Figure 6A, the synthesis of melanin is a complex process. The expression of melanin formation-related genes *VdT3HR*, *VdT4HR*, and *VdSCD* was significantly downregulated after treatment with strain T2 VOCs compared with the control levels. Among them, the relative expression level of the *VdSCD* exhibited the greatest downregulation (by approximately 29.41-fold), followed by that of *VdT4HR*, which was downregulated by approximately 10.49-fold, and the expression of *V3T4HR* was downregulated 3.11-fold (Figure 6B). As shown in Figure 6C, the melanin contents of *V. dahliae* after treatment with the T2 VOCs and individual commercial standards were significantly lower than that of the control; among these treatments, the VOC mixture exerted the strongest inhibitory effect, followed by 3-methyl-1-butanol, 3-octanone and butanoic acid, and ethyl ester, and 2-hexyl-furan exerted the weakest inhibitory effect. The results showed that the VOCs of strain T2 can inhibit the formation of melanin in *V. dahliae*.

## Discussion

To determine the antagonistic components in the VOCs of strain T2, six substances were detected using the SPME/GC–MS technique, and among these, 3-methyl-1-butanol, 3-octanone and butanoic acid ethyl ester exerted obvious effects on the mycelial growth, microsclerotia and melanin formation of *V. dahliae*. Notably, the VOCs of *T. koningiopsis* have been studied. The VOCs of *T. koningiopsis* PSU3-2 that exert an antagonistic effect are mainly azetidine, 2-phenylethanol and ethyl hexadecanoate, which can reduce the occurrence of chili pepper anthracnose caused by *C. gloeosporioides* (Ruangwong et al., 2021a). VOCs of *T. koningiopsis* T-51 showed inhibition of *B. cinerea* infection on tomato fruit (You et al., 2022). Several VOCs that were identified in our study are different from these above-mentioned compounds, and these differences may be due to the different habitats of these three strains: strains P3U3-2 and T-51 were isolated from the rhizosphere soil, whereas strain T2 was isolated from the leaves of *L. chinense* × *tulipifera* (Kong et al., 2022). These findings show that the secondary metabolites from microorganisms are rich and varied.

Previous studies have shown that 3-octanone produced by *Metarhizium anisopliae* can attract and kill plant parasitic nematodes (Hummadi et al., 2021). The VOCs produced by *Rahnella aquatilis* JZ-GX1 include 3-methyl-1-butanol, which inhibits the mycelial growth and spore germination of *C. gloeosporioides* and leads to mycelial disintegration (Kong et al., 2020b). The median effective dose of butanoic acid ethyl ester produced by *Candida intermedia* was 18.6 ± 6.4 μL/L against *Botrytis cinerea* growth (Suwannarach et al., 2016). These findings were consistent with our results. However, no related reports indicate that 2-hexyl-furan can be used as antifungal substances. We obtained the first demonstration that this compound can be used as effective chemical components to control *V. dahliae*, and this finding may provide new and improved microbial resources to control the disease.

The pathogenic mechanism of *V. dahliae* includes two theories, one of which is vascular bundle blockage theory (Ma et al., 2021). Studies have shown that *V. dahliae* has more enzymatic genes that degrade cell walls than other plant pathogenic fungi (Jeffress et al., 2020). This fact may explain why *V. dahliae* can remove the obstacles of some pectin substances in plants within a short time after inoculation, and this step is followed by colonization and diffusion in the xylem ducts. Simultaneously, different hydrolases can degrade the cell wall tissues of different host plants, which is one of the reasons why *V. dahliae* has a wide range of hosts (Lorrai and Ferrari, 2021). *V. dahliae* is rich in pectinase and endoglucanase, which can hydrolyze pectin and cellulose on the cell wall of host plants, and its degradation products can also be used as important nutrients to provide energy for growth and development (Safran et al., 2021). A previous study found that the pectinase gene VdPL1-4 can induce cell death and the plant defense response, and the deletion of VdPL1-4 can significantly reduce the virulence of strain Vd991 on cotton (Jiang et al., 2022). The endoglucanase gene *VdEg-1* is very important for early infection and colonization. The deletion of *VdEg-1* leads to poor pathogen colonization on lettuce (Maruthachalam et al., 2011). In this study, the VOCs produced by strain T2 significantly inhibited the activities of pectin lyase and endoglucanase of *V. dahliae*, which indicated that the application of strain T2 could reduce the early infection of *V. dahliae* on the host, and may subsequently decrease the occurrence of the disease.

The microsclerotia produced by *V. dahliae* is the main survival structure in soil and can survive in host-free soil for as long as 14 years (Van Eerd et al., 2015). A large amount of DHN melanin is attached to the intercellular space and cell wall of the microsclerotia, which can resist the effects of an adverse environment (Fan et al., 2017b). The traditional DHN melanin biosynthesis pathway includes four key enzymes, which are encoded by *VdPKS*, *VdT4HR*, *VdSCD*, and *VdT3HR*, and the expression of these genes is induced during the development of microsclerotia (Duressa et al., 2013; Xiong et al., 2014). Among these genes, deletion of the *VdT3HR* gene results in loss of the ability to form microsclerotia, and no microsclerotia formation was observed even after growth on medium that promotes microsclerotia production for 4 weeks. Knockout of the *VdT4HR* and *VdSCD* genes results in a colony with a dark-orange surface, which indicates a decrease in melanin production (Fan et al., 2017a). In this study, the VOCs produced by strain T2 decreased the expression of these three genes of *V. dahliae*, and the results from the melanin determination and microsclerotia observation support this conclusion. This pathway is very important for controlling plant *Verticillium* wilt caused by *V. dahliae*.

An important aspect that will require future studies is how to transfer all the results obtained in controlled laboratory conditions to plant disease management, several authors have reported differences in volatile interactions when they take place in closed or more open environments, there are many factors that can reduce or even eliminate the effects of these volatiles in the field (Alvarez-García et al., 2021, Álvarez-García et al., 2022). For instance, 2,3-butanediol released by *Bacillus subtilis* and *B. amyloliquefaciens* used under field conditions did not show its role as a plant growth regulator (Kanchiswamy et al., 2015). Another challenge is to assess the side effects of these volatiles, which are highly active and potentially hazardous. Some volatiles that are effective for use in plants have adverse side effects on non-target organisms such as insects, nematodes and humans.

Of course, the biocontrol mechanisms of *Trichoderma* are varied. Studies have shown that *T. koningiopsis* can also induce plant systemic resistance. Yu and Luo (2020) found that *T. koningiopsis* can control the death of Masson pine seedlings caused by *Fusarium oxysporum* by regulating active oxygen metabolism, osmotic potential and rhizosphere microorganisms. Whether strain T2 can also induce plant resistance to *Verticillium* wilt remains not clear. In addition, since the rapid growth rate of *Trichoderma* members preceded the formation of pathogen niche, the existence of hyperparasitism and the production of other non-volatile antagonistic substances, the biocontrol potential of strain T2 should be studied further. Therefore, there remains a lot of work and many challenges to overcome before strain T2 can be used to control plant *Verticillium* wilt, but the production of these VOCs are certainly more supportive of the biological control effect of strain T2.

## Conclusion

**FIGURE 7:**
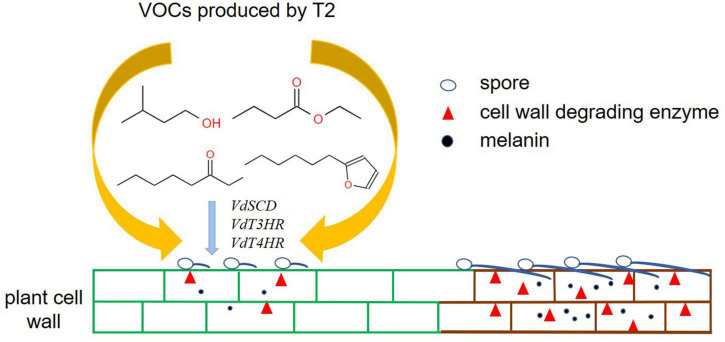
Hypothetical model of the mode of action of VOCs produced by *T. koningiopsis* T2 against *V. dahliae*.

In summary, this study provides the first demonstration that VOCs produced by *T. koningiopsis* T2 can directly inhibit the mycelial growth and microsclerotia formation of *V. dahliae* and significantly reduce the production of pectin lyase and endo-β-1,4-glucanase. In addition, the mixture of VOCs and individual commercial standards decrease the content of melanin and downregulate the expression of related genes, including *VdT3HR*, *VdT4HR*, and *VdSCD* (Figure 7). The potential to develop a biopesticide for the control of *Verticillium* wilt caused by *V. dahliae* using *T. koningiopsis* T2 must be verified in the future.

## Data availability statement

The original contributions presented in this study are included in the article/supplementary material, further inquiries can be directed to the corresponding author/s.

## Author contributions

W-LK and W-YW completed the experimental research. W-LK wrote the first draft of the manuscript. HN participated in the analyses of the experimental results. X-QW directed the experimental design, data analysis, and writing and revision of the manuscript. All authors read and agreed on the final text.
